# Supplementary material for: Life before Stonehenge: The hunter-gatherer occupation and environment of Blick Mead revealed by sedaDNA, pollen and spores
Source: PLoS One. 2022 Apr 27;17(4):e0266789. doi: 10.1371/journal.pone.0266789 (PMC9045597; doi:10.1371/journal.pone.0266789)
Supplement: S1 Text — (DOCX) [file pone.0266789.s001.docx]

S1 Text. Archaeological context

Archaeology

The Blick Mead site is divided into two parts: a wetter floodplain at the bottom the hollow, and a drier river terrace topped by lynchets. Numerous trenches have been excavated at the site, with Trenches 19, 22, 24, 28 and 31 containing significant Mesolithic archaeology.

The site has a complex hydrology, the earliest deposits are sands and gravels suggestive of a faster moving river formed on top of an Upper Chalk bedrock, likely in the Late Glacial period. Overlying these sands are a series of alluvial clays and silts that contain the Mesolithic horizons, these are between 0.2-0.44m thick and lithostratigraphic analysis has suggested they were deposited in a low energy environment of still or slow-moving water. The Mesolithic flints and animal bones found in this layer are thought to represent largely *in-situ* deposition. The prehistoric horizons are overlain and underlain with a cobbled flint surfaces, possibly deliberately constructed and of uncertain date. Further alluvial, peat and made ground horizons complete the top of the sequence.

The faunal remains at Blick Mead comprise of over 2400 animal bones, 271 identified to species (S3 Table). Auroch remains make up 57% of the identifiable assemblage, with almost all auroch remains coming from Trench 19 (along with the auroch hoofprints). Other large animal remains were less numerous, but consisted of red deer, elk, roe deer, wild boar and domestic dog, and from the upper terrace contexts- domestic pig, sheep/goat and rabbit, alongside smaller vertebrates and insects (S4 and S5 Tables).


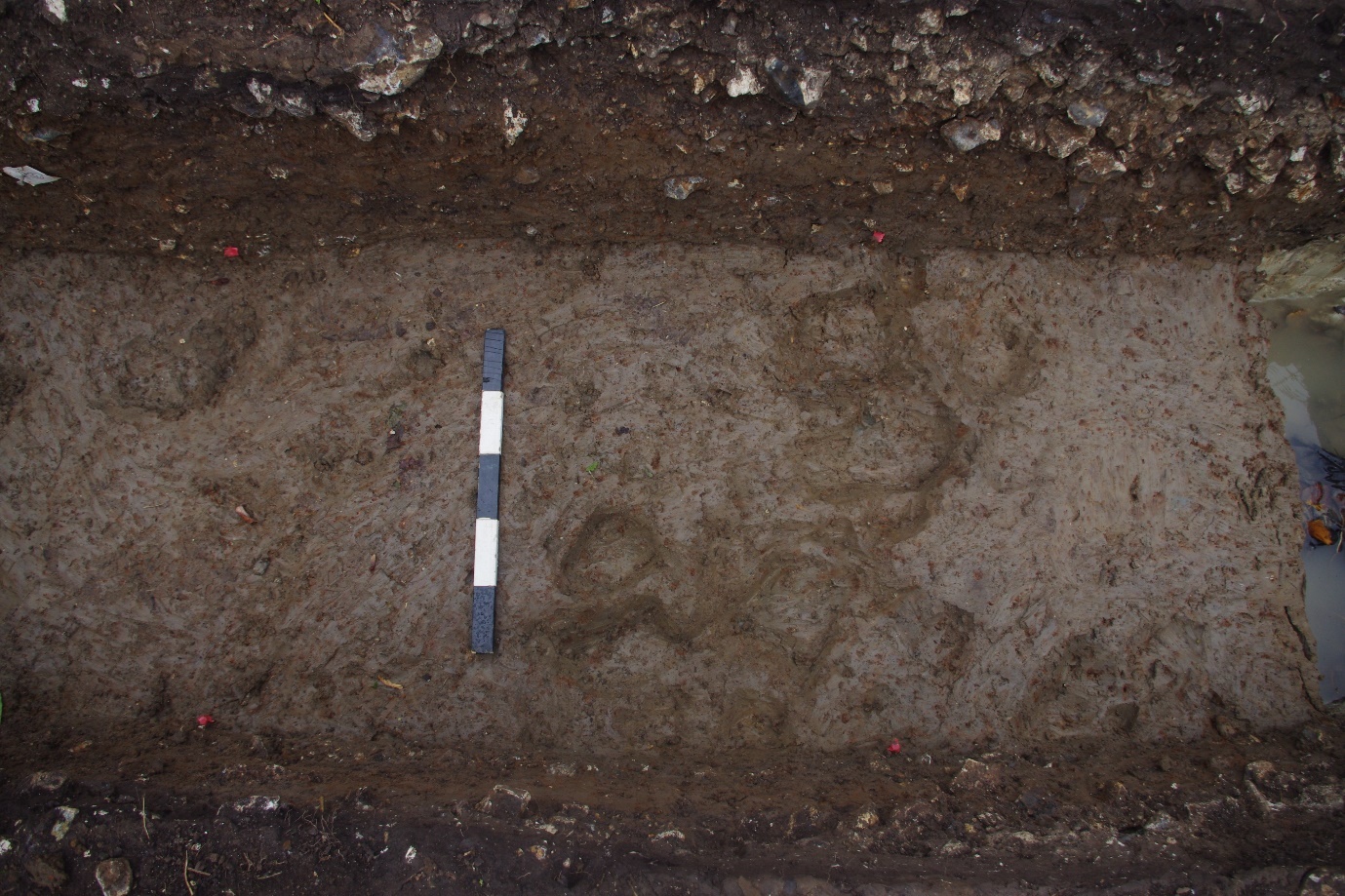


*Plate 1* Auroch hoofprints pressed into the alluvial clay Mesolithic horizons found in Trench 19.

The lithics from the site included over 100,000 pieces of struck flint, the majority came from the Mesolithic horizons from Trench 19 and is of Mesolithic date. Above the cobbled flint layer overlying the sampled contexts, further struck flint was encountered, much of it Mesolithic but also containing types typical of Neolithic and Bronze Age industries. The flintwork is for the most part, in a very good/sharp condition, suggesting little movement since deposition. It is thought that the flints themselves were knapped on the drier, higher ground of the terrace and then discarded into the wetland.

An additional unique feature of the Blick Mead site is the presence of a nearby spring-fed pond in the hollow which colours flint tools pink when left in the water for between 24-28 hours. This is due to presence of an algae known as *Hildenbrandia rivularis* which requires the 10-15 degree temperature maintained in the sheltered pond to persist. If this alga was present in the Mesolithic period (there is no reason to believe the spring water properties have changed significantly) then it could have been another attraction for Mesolithic exploitation of the area

Site Stratigraphy and Chronology

A borehole transect survey demonstrated an extensive area of interdigitated peat and silt dominated minerogenic alluvium (0.2-0.8m thick) at the base of the sequence which thinned away from the river and filled a small hollow 50m by 40m within the underlying reworked chalk and gravel superficial geology. No dating evidence was gathered from these deposits however the textural similarity and stratigraphic relationship between the minerogenic alluvium close to the river and adjacent to the sampling area suggests slow, regular alluviation around the same time, and that as a result the peats were at least Mesolithic in date and probably older.

The peat itself may have developed within a slight channel shift in the early Holocene, but no evidence of palaeochannels or former river movement was identified nearer the sample area or floodplain edge suggesting the archaeological site had never been directly adjacent to a major arm of the River Avon, although the remnant, highly disturbed depression immediately to the south may have at some time been an inferior backwater channel at a time when the watercourse had a higher level of bifurcation.

The borehole survey also highlighted the extent to which later human alterations to the floodplain had significantly altered the surface topography of the landscape. Across the majority of the cores up to a meter of chalky made-ground was identified increasing to the northeast. Two distinct phases of deposition could be seen intersected by clear buried topsoil ‘A’ horizons. The first of these was shallower (0.1-0.3m) and represented landscape garden engineering of Amesbury Abbey in the 18^th^ century. The second phase of made-ground accretion was thicker (0.6-0.8m) and more extensive and relates to the development of the current A303 main road in the 1960s.

From the OSL and radiocarbon dating, the top of the analysed sequence consisted of a Mesolithic flint stone platform within an organic rich soil (context 329) was dated to the Late Mesolithic (4236-4052 cal BCE) (all C^14^ dates listed as 95% confidence interval). Underlying the platform were the Mesolithic alluvial clays/silts, contexts 328 and 330. OSL samples within context 330 returned Late Mesolithic dates of (4000±330BCE) and (4910±420BCE). The reworked chalk beneath was dated using OSL to the Late Pleistocene (10,350±800BCE). The base of the lynchets overlying the analysed sequence was dated to the Middle Bronze Age (1433±150BCE).
