# Supplementary material for: Life before Stonehenge: The hunter-gatherer occupation and environment of Blick Mead revealed by sedaDNA, pollen and spores
Source: PLoS One. 2022 Apr 27;17(4):e0266789. doi: 10.1371/journal.pone.0266789 (PMC9045597; doi:10.1371/journal.pone.0266789)
Supplement: S2 Text — (DOCX) [file pone.0266789.s002.docx]

S2 Text. OSL Methodology.

OSL Methodology

The OSL dating undertaken for this study was part of a larger project to date the overlying terrace deposits alongside the Mesolithic horizons at Blick Mead, performed by Dr Andreas Lang and Dr Lisa Snape. The methodology is summarised here. Two grain-size fractions were investigated to determine the luminescence characteristics of quartz from the deposits sampled. Fine grains (4-11 μm) and coarse grains (90-125, 125-150 μm) were extracted from each sample. Quartz was isolated for equivalent dose (De) measurements following procedures outlined in Mauz et al., (2002) and Mauz and Lang (2004). Samples were wet-sieved through a 63 μm sieve to separate coarse from fine grains. Both fractions were chemically treated with hydrochloric acid (HCL) (10 and 37%) and hydrogen peroxide (H2O2) (10 and 30%). Fine grains were separated using the Atterberg separation technique to concentrate the 4-20 μm fraction. Coarse grains were etched with hydroflouric acid (HF) (47%) to remove the outer edge of grains and feldspars, followed by HCL (10%) to remove fluoric precipitates that can occur after HF treatment. Coarse grains were density separated using LST at 2.62 and 2.76 kg/ltr to separate quartz from feldspars and heavy minerals. The concentrated quartz fraction was then etched with HF and HCL. To derive fine silt-sized quartz the 4-20 μm polymineral fraction was etched with 20% for 20 minutes repeatedly until no significant IRSL signal could be observed.

To measure the De, small aliquots (sample covering ~1 mm diameter in the central area of a 10mm stainless steel cup) of coarse grains were mounted with silicon oil. For fine silt-sized quartz, sample material was mounted on 10 mm diameter stainless cups by settling ~2 mg of grains in deionised water.

OSL measurements were performed using a Lexsyg smart (Freiberg Instruments) equipped with blue LEDs (458 nm, 100 mW/cm2) and infrared (IR) LEDs (850 nm, 300 mW/cm2). OSL and IRSL signals were detected using a photomultiplier (PMT) tube with GG420 (3 mm) + HC 458/10 filters (blue) and RG 715 (3 mm) (IR) (Richter *et al*., 2015). RISØ filter combinations were used to detect blue stimulated luminescence (BSL) using a Hoya U340 7.5 mm, and IRSL was detected using a wideband blue filter.

The single-aliquot dose regenerative (SAR) protocol was employed (Murray and Wintle 2000; 2003). This involved measurement of the natural OSL signal followed by a series of regenerative doses. Two samples with a minor IRSL signal was measured using a post-IR blue SAR procedure A dose-recovery test was performed on each sample between 180-260 °C to determine the most appropriate preheat treatment during the SAR protocol (Murray and Wintle 2000, 2003). The following acceptance criteria were applied to each De measurement; (1) natural signal greater than ~200 counts per second (cps), (2) IR depletion and recycling ratios between 0.85 and 1.15, (3) and recuperation < 5% of the De signal. The fast ratio (FR) (Durcan and Duller 2011) was calculated for each aliquot to determine the presence of a fast dominating signal required for the SAR procedure to be performed correctly. De measurements were processed using Analyst software (v4.57). Blue OSL decay curves were measured by heating the aliquot with 5 °C to 125 °C held for 40 seconds and then cooled for 50 seconds made at 100 % stimulation power. For IRSL decay curves, the aliquot was heated with 5 °C to 50 °C and held for 100 seconds. The late background subtraction (LBS) was used. A post-IR blue SAR procedure (Mauz and Lang 2004, Shen et al., 2007) was applied to two samples with a minor IRSL signal. This was followed by a thermal stability test (Shen et al., 2011) to ensure that the pure OSL signal was pure. For estimation of the dose from the distribution of De values from fine silt-sized quartz was based on the arithmetric mean of the accepted aliquots. The relative error (RE) and skewness values were calculated for the accepted De values.

To determine the natural radioactivity of the samples, a subsample was dried at 50 °C for 24 hours to determine the water content (%), the same material was measured using a high-resolution low-level gamma spectrometer (HRGS). The dose rate was calculated using DRAC (v1.2) (Durcan *et al*., 2015). Radioactive isotope concentrations were converted to natural dose rate following Guerin *et al*., (2011) and water attenuation effect corrected following Aitken and Zie (1990) and Zimmerman (1971). Evidence for disequilibria was assessed for all samples, the maximum and minimum vales were used to assess the effects on OSL ages, their effects on the ages including errors were negligible. Only the lowermost sample (SBG140) had large errors and Uranium content had a significant effect on the OSL age. Cosmic dose rate was calculated following Prescott and Hutton (1994).

References

1. Aitken, M.J., Xie, J., 1990. Moisture correction for annual gamma dose. *Ancient TL* 8, 6-9.
2. Durcan, J.A. and Duller, G.A., 2011. The fast ratio: a rapid measure for testing the dominance of the fast component in the initial OSL signal from quartz. Radiation Measurements, 46(10), pp.1065-1072.
3. Durcan, J.A., King, G.E., Duller, G.A.T., 2015. DRAC: Dose rate and age calculator for trapped charge dating. *Quaternary Geochronology*, 28, 54-61.
4. Galbraith, R.F., Roberts, R.G., Laslett, G.M., Yoshida, H. and Olley, J.M., 1999. Optical dating of single and multiple grains of quartz from Jinmium rock shelter, northern Australia: Part I, experimental design and statistical models. *Archaeometry*, *41*(2), pp.339-364.
5. Guerin, G., Mercier, N., Adamiec, G., 2011. Dose-rate conversion factors: update. *Ancient TL*, 29, 5-8.
6. Mauz, B., Bode, T., Mainz, E., Blanchard, H., Hilger, W., Dikau, R. and Zöller, L., 2002. The luminescence dating laboratory at the University of Bonn: equipment and procedures. *Ancient TL*, *20*(2), pp.53-61.
7. Mauz, B. and Lang, A., 2004. Removal of the feldspar-derived luminescence component from polymineral fine silt samples for optical dating applications: evaluation of chemical treatment protocols and quality control procedures. *Ancient TL*, *22*(1), pp.1-8.
8. Murray, A.S. and Wintle, A.G., 2000. Luminescence dating of quartz using an improved single-aliquot regenerative-dose protocol. *Radiation measurements*, *32*(1), pp.57-73.
9. Murray, A.S. and Wintle, A.G., 2003. The single aliquot regenerative dose protocol: potential for improvements in reliability. *Radiation measurements*, *37*(4-5), pp.377-381.
10. Prescott, J.R. and Hutton, J.T., 1994. Cosmic ray contributions to dose rates for luminescence and ESR dating: Large depths and long-term time variations. *Radiation Measurements*, 23, 497-500.
11. Richter, D., Richter, A. and Dornich, K., 2015. Lexsyg smart—a luminescence detection system for dosimetry, material research and dating application. *Geochronometria*, *42*(1).
12. Zimmerman, J., 1971. The radiation-induced increase of the 100 C thermoluminescence sensitivity of fired quartz. *Journal of Physics C: Solid State Physics*, *4*(18), p.3265.
