# Supplementary material for: Life before Stonehenge: The hunter-gatherer occupation and environment of Blick Mead revealed by sedaDNA, pollen and spores
Source: PLoS One. 2022 Apr 27;17(4):e0266789. doi: 10.1371/journal.pone.0266789 (PMC9045597; doi:10.1371/journal.pone.0266789)
Supplement: S3 Text — (DOCX) [file pone.0266789.s003.docx]

# S3 Text- Extended Micromorphology Results

The micromorphological results from Blick Mead demonstrate depositional conditions associated with low-energy fluvial conditions. At the base of the sequence the reworked chalk (334) had a dominance of calcite, silicates and chalk with a distinctive grey to dark grey colour with specked to cloudy limpidity, a massive microaggregate structure with random and well-sorted coarse mineral arrangement and a crystallic groundmass ‘b’ fabric and a close porphyric related distribution, typical of carbonate geologies. Coarse and fine organics were not present and no evidence of pedofeatures, although there were amorphous black and orange material, likely minerogenic, and infrequent small iron minerals, all likely derived from groundwater translocation.

Above the chalk, with a sharp boundary, was a distinctive light yellowish brown coloured horizon (330) which also demonstrated a massive microaggregate structure and well-sorted coarse mineral arrangement. This context contained lower levels of calcite, chalk and flint but slightly increased manganese and glauconite typical of greater fine sediment fractions. There was also a shift to a stipped to flecked groundmass ‘b’ fabric and change to a double spaced fine enaulic to single spaced porphyritic related distribution, representing a shift to lower energy depositional conditions. The greatest shift was seen in the frequency of coarse and fine organic inclusions including macro plant fragments and degraded organics, alongside greater micro amorphous black, brown and orange material associated with progressive degradation over time. Pedofeatures also increased and included greater numbers of silt and clay void infills, organic coatings and consistent iron nodules.

Between horizons (330) and the above horizon (328) was a graded boundary with a subtle textural change. The horizon had a darker yellowish grey brown colour with a distinguishable increase in coarser quartz and flint inclusions and a major reduction in macro calcite and chalk. Structurally though the deposit still presented evidence of a predominantly fine intergrain to massive microstructure, spotted/specked limpidity, stippled/flecked groundmass ‘b’ fabric and shift to a single spaced porphyritic and chitonic structure distribution. Alongside greater frequency of coarser mineral inclusions the horizon also demonstrated increased coarse and fine organics including distinguishable charcoal, high levels of plant macrofossils and trace evidence of lignified tissue, spores and turf alongside consistent frequencies of amorphous black, brown and orange fine organics but slightly lower levels of silt and clay void infills, and considerably lower iron nodules.
