# Supplementary material for: Life before Stonehenge: The hunter-gatherer occupation and environment of Blick Mead revealed by sedaDNA, pollen and spores
Source: PLoS One. 2022 Apr 27;17(4):e0266789. doi: 10.1371/journal.pone.0266789 (PMC9045597; doi:10.1371/journal.pone.0266789)
Supplement: S1 Table — (DOCX) [file pone.0266789.s004.docx]

| Trench | Context no./ depth (m OD) | Feature/ Context type | Material | Lab no. | Radiocarbon age BP | Cal bc (68%) | Cal bc (95%) |
| --- | --- | --- | --- | --- | --- | --- | --- |
| 24 | [221] | Tree throw [105], lower filI | Wood charcoal | SUERC-66824 | 8775 ±29 | 7937–7751 | 7960–7716 |
| 19 | [67] | Layer [59] | Bovine tooth | SUERC-42525 | 8542 ± 27 | 7593–7569 | 7596–7542 |
| 23 | [90] | Layer [90] | Aurochs | SUERC-51968 | 7808 ± 33 | 6657–6600 | 6698–6531 |
| 19 | [65] | Layer [59] | Aurochs | SUERC-33649 | 7355 ± 30 | 6330–6100 | 6360–6080 |
| 19 | [92] | Layer [59] | Red deer | SUERC-51973 | 7294 ± 34 | 6214–6106 | 6226–6074 |
| 19 | [59c] | Layer [59] | Aurochs | SUERC-60917 | 6881±33 | 5793–5723 | 5845–5686 |
| 19 | [64] | Layer [59] | Wild pig | SUERC-42341 | 6396 ± 26 | 5464–5325 | 5469–5320 |
| 22 | [91] | Layer [91] | Aurochs | SUERC-51969 | 6198 ± 32 | 5216–5073 | 5289–5048 |
| 19 | [77.1] | Layer [59] | Aurochs | SUERC-47248 | 6114 ± 28 | 5199–4992 | 5208–4948 |
| 19 | [76] | Layer [59] | Aurochs | SUERC-46224 | 6018 ± 31 | 4947–4848 | 4998–4810 |
| 19 | [77.5] | Layer [59] | Red deer | SUERC-51972 | 6009 ± 28 | 4939–4848 | 4989–4808 |
| 19 | [67] | Layer [59] | Aurochs | SUERC-37208 | 5900 ± 35 | 4798–4722 | 4846–4695 |
| 19 | [77.4] | Layer [59] | Aurochs | SUERC-51971 | 5881 ± 26 | 4781–4722 | 4826–4702 |
| 24 | [107] | Layer – loess | Oak charcoal | SUERC-66820 | 5412±30 | 4327–4259 | 4340–4183 |
| 24 | [114] | Posthole [115] | Charcoal | SUERC-56919 | 5424 ± 25 | – | 4336–4246 |
| 24 | [212W] | Tree throw [111], lower fill | Oak charcoal | SUERC-66822 | 5298 ± 30 | 4227–4051 | 4236–4041 |
| 24 | [218] | Tree throw [111], lower fill | Oak charcoal | SUERC-66823 | 5302 ± 29 | 4227–4054 | 4234–4045 |
| 24 | [210] | Tree throw [111], main fill | Alder/hazel | SUERC-66821 | 4748 ± 29 | 3632–3520 | 3636–3381 |
| 24 | [314] | Flint platform above buried soil [329] | Oak Charcoal | SUERC-76890 | 5321 ± 24 | 4232-4070 | 4236-4052 |

# S1 Table

**Radiocarbon Dates from Blick Mead *dates already obtained from macrofossil and faunal remains. Taken from both the wetland site (Trench 19) and drier levels (trench 22, 23, 24). Adapted from Jacques et al. (2018).***
