# Supplementary material for: Life before Stonehenge: The hunter-gatherer occupation and environment of Blick Mead revealed by sedaDNA, pollen and spores
Source: PLoS One. 2022 Apr 27;17(4):e0266789. doi: 10.1371/journal.pone.0266789 (PMC9045597; doi:10.1371/journal.pone.0266789)
Supplement: S2 Table — (DOCX) [file pone.0266789.s005.docx]

S2 Table

***OSL dating results. SBG140 and SBG 141 are both from the sampling sondage within trench 31 where the DNA and sedimentological samples were taken. Samples SBG077, SBG080 and SBG086 were located within trench 24 and were targeted at the terrace deposits.***

| OSL sample | Quartz grain size | Natural dose rate    Gy/ka | Number of aliquots | Dose model | Skew | Overdispersion (%) | Relative error (%) | D  e  Gy | OSL age | Calendrical years  BCE/CE |
| --- | --- | --- | --- | --- | --- | --- | --- | --- | --- | --- |
| SBG077 | 4-11 | 2.10±0.12 | 19 | Mean | 0.16 | 1.14 | 0.79 | 13.31±0.11 | 6.67±0.29 | 4650±290 BCE (Late Mesolithic) |
| SBG080 | 4-11 | 1.86±0.11 | 22 | Mean | 2.49 | 1.56 | 0.87 | 5.71±0.05 | 3.19±0.17 | 1170±170 BCE  (Middle Bronze Age) |
| SBG086 | 4-11 | 1.44±0.11 | 17 | Mean | 2.92 | 5.92 | 1.71 | 4.82±0.08 | 3.46±0.64 | 1440±640 BCE  (Middle Bronze Age) |
| SBG140 | 4-11 | 1.17±0.08 | 22 | Mean | -  0.53 | 3.72 | 1.89 | 14.47±0.27 | 11.18±1.22 | 9160±1220 BCE Pleistocene |
| SBG141 | 4-11 | 2.29±0.14 | 27 | Mean | 0.25 | 0 | 0.95 | 15.90±0.10 | 7.71±0.67 | 5690±670 BCE (Late Mesolithic) |
