# Supplementary material for: Life before Stonehenge: The hunter-gatherer occupation and environment of Blick Mead revealed by sedaDNA, pollen and spores
Source: PLoS One. 2022 Apr 27;17(4):e0266789. doi: 10.1371/journal.pone.0266789 (PMC9045597; doi:10.1371/journal.pone.0266789)
Supplement: S3 Table — (DOCX) [file pone.0266789.s006.docx]

S3 Table

***The large vertebrate analysis from Blick Mead. The totals are numbers of identified specimens (NISP)* (Jacques *et al.* 2018).**

| **Species** | **Total** | **Trench 19** | **Trench 22** | **Trench 23** | **Trench 24** |
| --- | --- | --- | --- | --- | --- |
| Aurochs, *Bos primigenius* | 155 | 139 | 13 | 1 | 2 |
| Aurochs? cf. *Bos primigenius* | 4 | 4 | 0 | 0 | 0 |
| Red Deer, *Cervus elaphus* | 46 | 42 | 2 | 2 | 0 |
| Red Deer? cf. *Cervus elaphus* | 4 | 4 | 0 | 0 | 0 |
| Elk, *Alces alces* | 5 | 5 | 0 | 0 | 0 |
| Elk? cf. *Alces alces* | 1 | 1 | 0 | 0 | 0 |
| Red Deer or Elk, *Cervus* or *Alces* | 2 | 2 | 0 | 0 | 0 |
| Roe Deer, *Capreolus capreolus* | 8 | 7 | 0 | 1 | 0 |
| Wild Boar, *Sus scrofa ferus* | 23 | 23 | 0 | 0 | 0 |
| Sheep/Goat, *Ovis* or *Capra* | 9 | 3 | 5 | 0 | 1 |
| Domestic Cow, *Bos primigenius taurus* | 5 | 1 | 0 | 0 | 4 |
| Domestic Pig, *Sus scrofa domesticus* | 5 | 4 | 0 | 0 | 1 |
| Bird, *Aves* | 1 | 0 | 0 | 1 | 0 |
| Dog, *Canis familiaris* | 1 | 1 | 0 | 0 | 0 |
| Rabbit, *Oryctolagus cuniculus* | 2 | 0 | 0 | 0 | 2 |
| Total identified | 271 | 236 | 21 | 5 | 9 |
| Unidentified | 2159 | 2065 | 44 | 11 | 39 |
| **Total** | **2430** | **2301** | **65** | **16** | **48** |
