# Supplementary material for: Life before Stonehenge: The hunter-gatherer occupation and environment of Blick Mead revealed by sedaDNA, pollen and spores
Source: PLoS One. 2022 Apr 27;17(4):e0266789. doi: 10.1371/journal.pone.0266789 (PMC9045597; doi:10.1371/journal.pone.0266789)
Supplement: S4 Table — (DOCX) [file pone.0266789.s007.docx]

S4 Table

***The small vertebrate analysis from Blick Mead trench 19, contexts 74 and 59 subsampled. Counts represent the number of identified specimens (NISP) (Jacques et al. 2018).***

| Table 4 | **Trench 19 (Context)** | **[74]** | **[59]** | **[59]** | **[59]** | **[59]** | **[59]** | **[59]** | **[59]** | **[59]** | **[59]** |
| --- | --- | --- | --- | --- | --- | --- | --- | --- | --- | --- | --- |
|  | **Sub-sample no.** |  | **92.17** | **92.4** | **92.16** | **77.1** | **92.3** | **92.11** | **92.12** | **92.10** | **92.14** |
| Fish | Salmonid and pike |  | 6 | 3 | 3 | 1 | 3 | 4 | 4 | 3 | 3 |
| Anuran | Frog and/or toad |  | 1 | 2 |  | 1 | 1 |  |  |  |  |
| *Lacerta* sp. | Lizard |  |  |  |  |  |  |  |  | 1 |  |
| **Insectivore** |  |  |  |  |  |  |  |  |  |  |  |
| *Talpa europaea* | Mole |  |  |  | 2 |  |  |  |  |  |  |
| *Sorex araneus* | Common shrew |  |  |  |  |  |  |  | 1 |  |  |
| **Rodent** |  |  |  |  |  |  |  |  |  |  |  |
| *Clethrionomys glareolus* | Bank vole | 1 | 19 | 9 | 4 | 8 | 4 | 4 | 3 | 4 | 4 |
| *Microtus* sp. | Vole |  | 2 |  | 1 |  | 2 |  | 1 |  |  |
| *Arvicola terrestris* | Water vole |  | 1 | 1 | 2 |  | 2 |  |  | 1 |  |
| Microtine rodent | Vole |  | 4 | 1 | 1 |  |  | 2 | 3 | 4 | 1 |
| *Apodemus sylvaticus* | Wood mouse |  | 1 |  |  |  |  |  |  |  |  |
| *Apodemus flavicollis* | Yellow-necked mouse |  | 3 |  |  | 1 |  | 1 |  |  | 1 |
| *Apodemus* sp. | Mouse | 1 | 2 |  |  | 1 |  |  |  | 1 |  |
| Indet rodent |  | 3 | 12 | 8 | 9 | 5 | 5 | 5 | 4 |  | 4 |
| Small mammal |  |  | 1 |  |  |  |  | 1 |  |  |  |
| **Carnivore** |  |  |  |  |  |  |  |  |  |  |  |
| *Martes* sp. | Marten |  | 1 |  |  |  |  |  |  |  |  |
| **TOTAL (NISP)** |  | 5 | 53 | 24 | 22 | 17 | 17 | 17 | 16 | 14 | 13 |

| Table 4 cont. | **Trench 19 (Context)** | **[59]** | **[59]** | **[59]** | **[59]** | **[59]** | **[59]** | **[59]** | **[59]** | **[59]** | **[59]** |
| --- | --- | --- | --- | --- | --- | --- | --- | --- | --- | --- | --- |
|  | **Sub-sample no.** | **92.2** | **92.15** | **92.6** | **92.8** | **92.13** | **92.18** | **92.5** | **92.1** | **92.9** | **92.7** |
| Fish | Salmonid and pike | 1 | 2 |  |  |  | 2 | 1 |  |  |  |
| Anuran | Frog and/or toad |  |  |  | 1 |  |  |  | 1 |  |  |
| *Lacerta* sp. | Lizard |  |  |  |  |  |  |  |  |  |  |
| **Insectivore** |  |  |  |  |  |  |  |  |  |  |  |
| *Talpa europaea* | Mole |  |  |  |  |  |  |  |  |  |  |
| *Sorex araneus* | Common shrew |  |  |  |  |  |  |  |  |  |  |
| **Rodent** |  |  |  |  |  |  |  |  |  |  |  |
| *Clethrionomys glareolus* | Bank vole | 4 | 2 | 5 | 2 | 2 |  | 1 |  | 2 | 2 |
| *Microtus* sp. | Vole | 2 |  |  |  |  |  |  |  |  |  |
| *Arvicola terrestris* | Water vole | 1 |  |  |  | 1 |  |  |  |  |  |
| Microtine rodent | Vole | 2 | 2 |  |  |  |  |  | 1 |  |  |
| *Apodemus sylvaticus* | Wood mouse |  |  |  |  |  |  |  | 1 |  |  |
| *Apodemus flavicollis* | Yellow-necked mouse | 1 |  |  |  |  |  |  |  |  |  |
| *Apodemus* sp. | Mouse |  |  |  |  |  |  |  |  |  |  |
| Indet rodent |  | 1 | 5 | 4 | 2 | 1 | 2 | 1 |  | 1 |  |
| Small mammal |  |  |  |  | 1 | 1 |  |  |  |  | 1 |
| **Carnivore** |  |  |  |  |  |  |  |  |  |  |  |
| *Martes* sp. | Marten |  |  |  |  |  |  |  |  |  |  |
| **TOTAL (NISP)** |  | 12 | 11 | 9 | 6 | 5 | 4 | 3 | 3 | 3 | 3 |
