# Supplementary material for: Life before Stonehenge: The hunter-gatherer occupation and environment of Blick Mead revealed by sedaDNA, pollen and spores
Source: PLoS One. 2022 Apr 27;17(4):e0266789. doi: 10.1371/journal.pone.0266789 (PMC9045597; doi:10.1371/journal.pone.0266789)
Supplement: S5 Table — (DOCX) [file pone.0266789.s008.docx]

S5 Table

***The insect analysis from Trench 19, context 77 (Jacques et al. 2018).***

| **Taxon** | **Trench 19** | | |  |
| --- | --- | --- | --- | --- |
|  | **<5> (77.1)** | **<6> (77.3)** | **<7> (77.5)** |  |
| DYTISCIDAE (predaceous diving beetles) | | | | |
| *Agabus* sp. | 1 | - | 1 | - |
| HYDROPHILIDAE (Water scavenger beetles) | | | | |
| *Ochthebius minimus* (F.) | - | 1 | - | - |
| *Laccobius minutus* (F.) | 1 | - | - | - |
| *Enochrus* sp. | - | 1 | - | - |
| *Helophorus brevipalpis* Bedel | 1 | - | - | - |
| STAPHYLINIDAE (rove beetles) | | | | |
| *Lathrobium* sp. | 1 | 1 | - | - |
| *Tachyporus nitidulus* (F.) | 1 | - | - | - |
| CHRYSOMELIDAE (leaf beetles) | | | | |
| *Altica* sp. | 1 | - | - | - |
| APIONIDAE (seed weevils) | | | | |
| *Protapion apricans* Herbst | 1 | - | - | - |
| CURCULIONIDAE (weevils) | | | | |
| Genus et sp. indet. | - | - | - | 1 |
| HYMENOPTERA – FORMICIDAE (Ants) | | | | |
| *Myrmica schencki* Emery | 1 | - | - |  |
