# Supplementary material for: Life before Stonehenge: The hunter-gatherer occupation and environment of Blick Mead revealed by sedaDNA, pollen and spores
Source: PLoS One. 2022 Apr 27;17(4):e0266789. doi: 10.1371/journal.pone.0266789 (PMC9045597; doi:10.1371/journal.pone.0266789)
Supplement: S7 Table — (DOCX) [file pone.0266789.s010.docx]

| **Blick Mead, Wilts** | | | Coarse Rock & Mineral  Components (200 to <10µm)  (1) |  | Coarse Organics  (1) | Fine Organics  (1) | Pedofeatures  (2) |  |  |  | |  | |
| --- | --- | --- | --- | --- | --- | --- | --- | --- | --- | --- | --- | --- | --- |
| Horizon | | Depth (cm) | Calcite  Quartz  Chalk  Flint  Manganese  Olivine  Glauconite | Fine Mineral | Charcoal  Plant Fragments  Organics  Turf  Lignified Tissue  Spores | Amorphous Black  Amorphous Brown  Amorphous Orange  Cell Residue  Ash | Silt Infills  Clay Infills  Organic Coatings  Excremental Features  Iron Oxide Nodules | Microstructure | Coarse Material Arrangement | | Groundmass 'b' Fabric | | Related  Distribution |
| 328 | 2-10 | | *****  *******  *******  *****  **Trace**  ****** | Dark yellowish grey brown colour with spotted/speckled limpidity | *****  *******  ******  **Trace**  **Trace**  **Trace** | ******  ******  *******  **Trace** | *****  *****  *****  ***** | Intergrain to massive microaggregate structure | Random, with well sorted microstructure | Stippled, Flecked | | Single spaced porphyric to chitonic structure | |
| 330 | 16-24 | | ******  *******  ******  *****  ******  *****  ****** | Light yellowish brown colour with speckled limpidity | *******    ******* | ******  ******  ****** | ******  ******  *****  ******* | Massive microaggregate structure | Random, with well sorted microstructure | Stippled, Flecked | | Double spaced fine enaulic to single spaced porphyric structure | |
| 334 | 28-36 | | ********  *******  ********  ******  *****  ****** | Grey to dark grey colour with speckled/cloudy limpidity |  | *****  ****** | ******* | Massive microaggregate structure | Random, with well sorted microstructure | Crystallitic | | Close porphyric structure | |

(1) Frequency levels for coarse mineral components (Bullock *et al.* (1985) * very few, ** few, *** frequent/common, **** dominant/very dominant

(2) Frequency level for textural pedofeatures (Bullock *et al.* 1985) * rare, ** occasional, *** many

S7 Table, Summary of micromorphological results from Blick Mead, Wiltshire.
