# Supplementary figures and images for: Life before Stonehenge: The hunter-gatherer occupation and environment of Blick Mead revealed by sedaDNA, pollen and spores
Source: PLoS One. 2022 Apr 27;17(4):e0266789. doi: 10.1371/journal.pone.0266789 (PMC9045597; doi:10.1371/journal.pone.0266789)

S1 Figure

**Locations mentioned in text outside of the Stonehenge environs.**

**
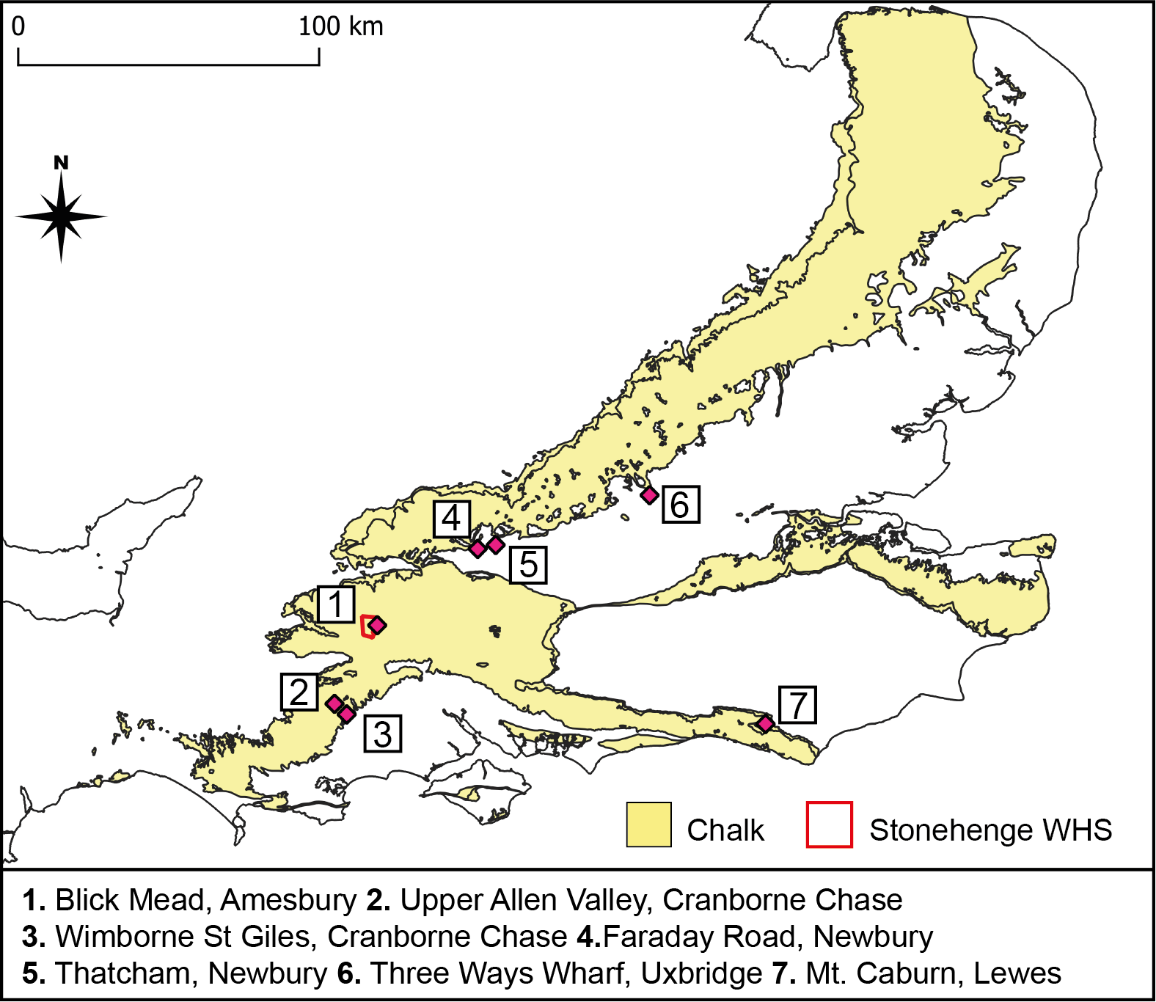
**

Supplement: S1 Fig — (DOCX) [file pone.0266789.s011.docx]

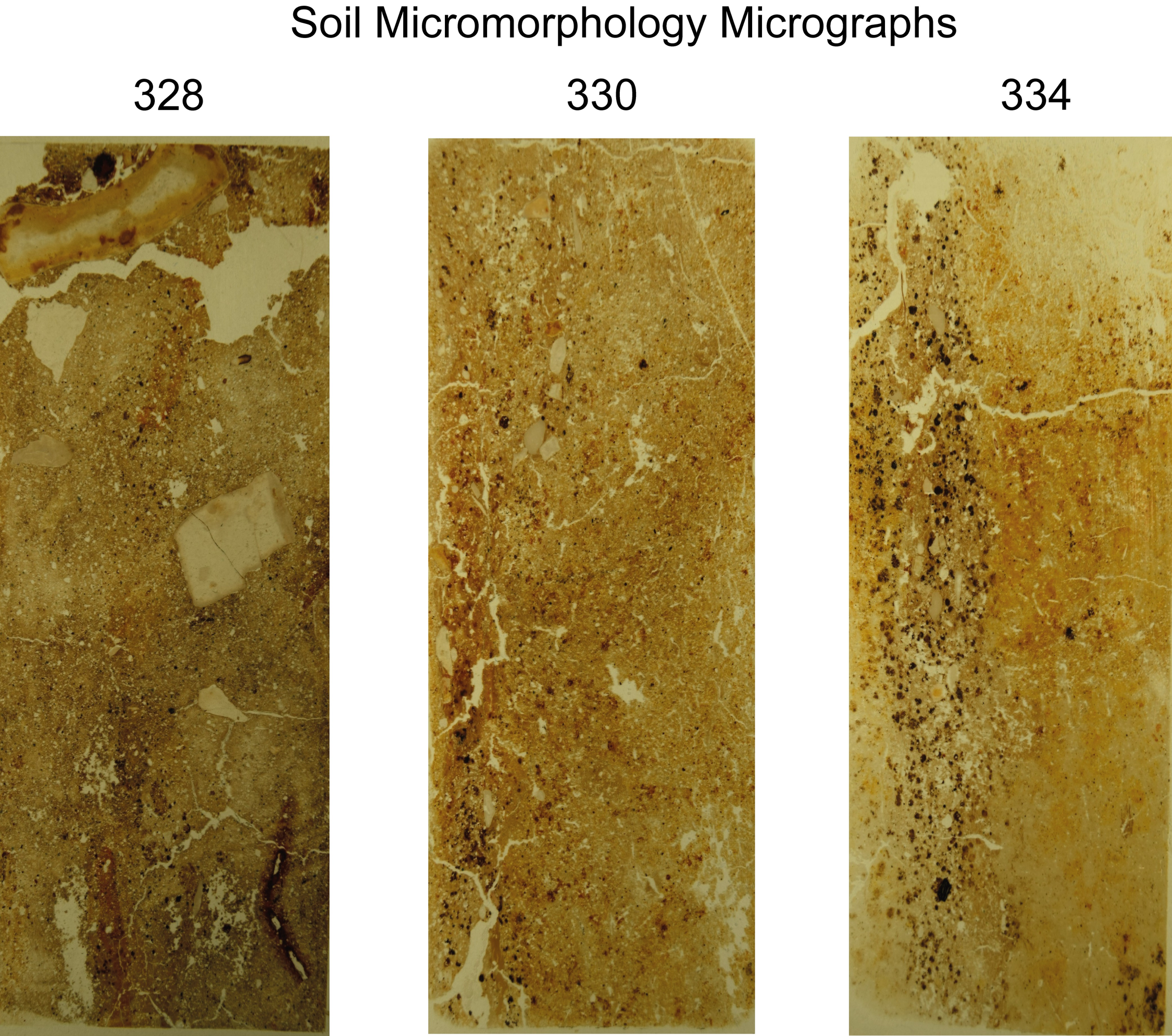

Supplement: S3 Fig — (TIF) [file pone.0266789.s013.tif]
