# Supplementary material for: Life before Stonehenge: The hunter-gatherer occupation and environment of Blick Mead revealed by sedaDNA, pollen and spores
Source: PLoS One. 2022 Apr 27;17(4):e0266789. doi: 10.1371/journal.pone.0266789 (PMC9045597; doi:10.1371/journal.pone.0266789)
Supplement: S2 Fig — (DOCX) [file pone.0266789.s012.docx]

S2 Figure

**Supporting LOI, XRF and Magnetic susceptibility data from the analysed 42cm sequence of the DNA and OSL sampling section within trench 31.**

**
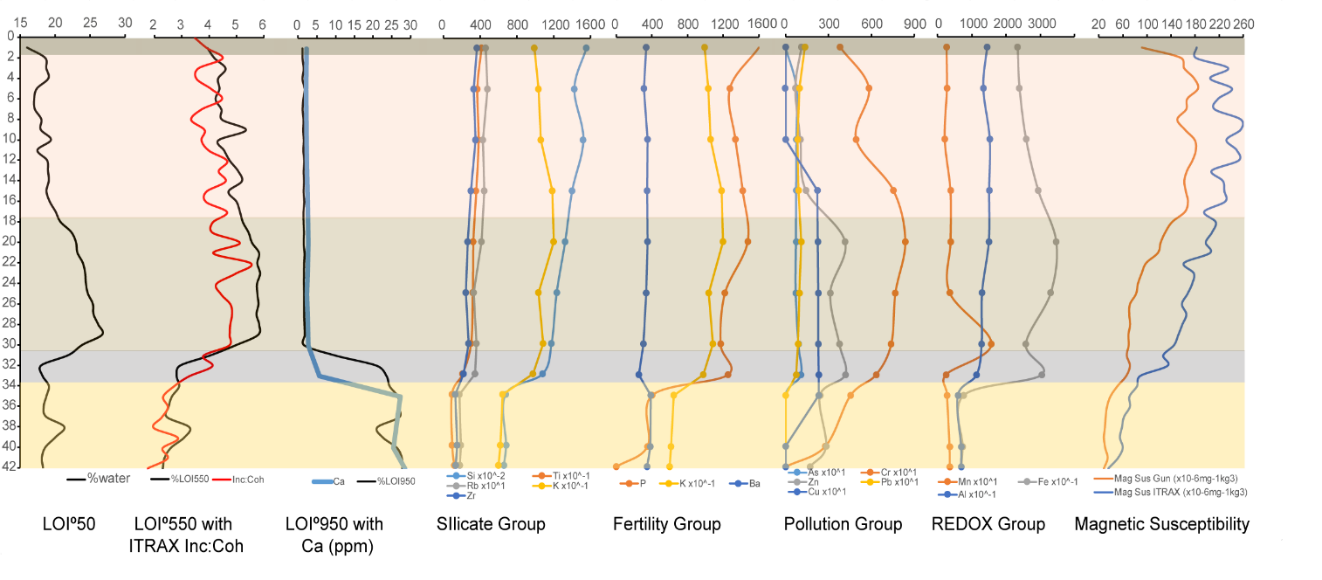
**
