# Supplementary material for: Life before Stonehenge: The hunter-gatherer occupation and environment of Blick Mead revealed by sedaDNA, pollen and spores
Source: PLoS One. 2022 Apr 27;17(4):e0266789. doi: 10.1371/journal.pone.0266789 (PMC9045597; doi:10.1371/journal.pone.0266789)
Supplement: Attachment — Submitted filename: Response to Reviewers.docx [file pone.0266789.s017.docx]

**Dear Peter F. Biehl (Academic Editor),**

**We here submit the revision of the paper “*Life before Stonehenge: the hunter-gatherer occupation and environment of Blick Mead revealed by sedaDNA, pollen and spores.”***

**Can we thank the referees for their valuable comments almost all of which we have acted upon and revised the paper accordingly. Below we provide a point-by-point response to the comments and also have included these comments into the revised tracked changes version of the paper – along with the revised non-tracked changes version. The major point about mixing of the Mesolithic horizons is valid and we have following the referees comments now provided some soil micromorphology and further comments regarding both the chronology and the hydromorphic nature of the sediments which we think improves the paper. We have made all the minor changes the referees suggested.**

**We hope these changes meet with both the referees’ and your approval.**

**Best wishes,

Sam Hudson**

**Responses to Referees Comments and Actions Taken (in bold)**

1. Line 125-128: “Certainly, the presence of unusually high prominence of auroch artefacts in the macrofaunal assemblage, some with physical evidence of butchery, alongside numerous auroch hoofprints identified within Mesolithic soil horizons, suggests a heightened exploitation of this particular animal at Blick Mead [Line 125-128]
   COMMENT: Cut-marked bones resulting from butchery are not normally considered “artefacts”. Why not simply refer to them as “cut-marked bones”? It is difficult to see how hoofprints are necessarily linked to human exploitation, or the combination of a few cut-marked bones plus hoofprints (especially given the 3-4000 yr Mesolithic occupation history) is indicative of “heightened exploitation”. **Agreed. Actions taken: We have removed the term “artefacts” and the term ‘heightened’.**

   2. Line 206-208: “The late Mesolithic [OSL] dates for the middle of the sequence align with late Mesolithic C14 dates from nearby trenches 19, 22 and 24”.
   COMMENT: The 2-sigma errors on the OSL dates are very large (up to ±1280 yr). So, “align” would seem to be an overstatement. **Agreed, although the maximum error range for the Late Mesolithic contexts is ±670yrs at 2σ . Actions taken: have altered the sentence to “The late Mesolithic dates for the middle of the sequence overlap with late Mesolithic C14 dates from nearby trenches 19, 22 and 24”.**

   3. Line 210-212: “… reinforcing the relative archaeological chronology provided by the stone tool assemblage and demonstrating evidence of no obvious, major contamination of DNA results from later human activity”.
   COMMENT: What is meant by “relative archaeological chronology”. Was this established from stone artefact typology or stratigraphy or both, and when and by whom; moreover, how does this “demonstrate” the stratigraphic integrity of the sedaDNA results – give reference citation(s). I don’t have access to the 2018 monograph on Blick Mead (Ref. 2), but in his review of that monograph report Andrew David (whose opinion I value) states, “What is clear, but is perhaps not spelled out enough in the accompanying discussion, is that the Mesolithic deposit is a chronologically mixed accumulation” (David 2019: 440). This apparent contradiction needs to be addressed. **This view was based on research up to 2013. Since then further stratigraphic work has established a tighter stratigraphy in Trenches 24, 24c and 31 (where our DNA samples were located) from stone artefact typology, sediment stratigraphy. Actions taken: we have included further information regarding the stone artefact typology and chronological implications from Trench 24, referenced by personal communication with the lithics specialist from Blick Mead- Barry Bishop on the 26/01/22**

   4. Line 331: “There were three intrinsic measures of sedaDNA authenticity for this site … [et seq.]”
   COMMENT: The three reasons given for discounting vertical translocation of sedaDNA are observed differences between the (sedaDNA-derived) plant assemblages from the Mesolithic age (alluvial) sediments and the underlying weathered chalk (1) and the modern flora (2), and colluviation (linked to Bronze Age (BA) agriculture on the adjacent terrace) protecting the Mesolithic horizons from later disturbance (3). None of these is entirely convincing. BA and later colluviation might explain the absence of modern plant DNA but would not have protected the Mesolithic horizons from pedogenic and other ‘disturbances’ during the Late Mesolithic to MBA (c. 5700-1500 BCE). From the context/horizon descriptions in Fig. 3A, I suspect the alluvial sediments were strongly affected by pedogenesis over a prolonged period, and that more-or-less the entire profile (from context 329 downwards into 334) is a buried soil. To be blunt, stratigraphic interpretation of palynological and sedaDNA data from buried soils is arguably no more reliable than from a modern soil. Micromorphological (thin section) analysis is widely used to investigate the properties and processes in buried soils/sediments, and often essential for identifying the extent of translocation due to root penetration, percolation, and (perhaps relevant here) animal/human trampling. Was soil microscopy and micromorphology applied at Blick Mead? If so, the results should have been included in this report. If not, this is a serious omission, which needs to be rectified before publication of the sedaDNA and palynological data. **The upper buried soil is we believe still stratified with minimal mixing due to waterlogging typical of hydromorphic soils and we have micromorphology to back this up. Actions taken: we have included a new paragraph and soil micromorphology figure (Fig 3) in the results along with a summary in the discussion and extended information in the S3 text and the S7 table. The soil micromorphology suggested sediment deposition in a low-energy, fluvial environment with no evidence of translocation between the horizons and minimal bioturbation.**

   5. METHODS/SUPPORTING INFORMATION: Overall, the descriptions of materials and methods are adequate, except for sediment geochemistry and particularly XRF. The micro-XRF (iTRAX) system is described in reference 69 (which is acceptable), but more explanation should be provided for the handheld pXRF measurements. Why was it deemed necessary to use both pXRF and μXRF? Were the pXRF measurements taken in the field or in the lab and, if in the lab, how were the samples prepared? What settings were used with the Niton XL3t – calibration model, filters, spot size, measurement times, etc. What are the detection limits for different elements using those settings? Was an empirical calibration used based on CRMs, or just the values provided by the instrument without external calibration? **Agreed. Actions taken: we have expanded the details on the pXRF methodology giving the detail requested and explained why we used both pXRF and μXRF.**

MINOR COMMENTS

General – “auroch” or “aurochs” are (alternative) SINGULAR nouns for Bos primigenius; “aurochsen” is the PLURAL/collective noun; **Agreed. Action taken: all plurals changed**
Line 19 – missing in Tromsø; **Agreed. Action taken: ‘o’ changed to ‘ø’**
Line 37 – “drylands” is one word; **Agreed**. **Action taken: all cases altered to one word**
Line 40 – “open clearing” – the word “open” seems superfluous; **Agreed. Action taken: Removed**
Line 59 – DELETE “over approximately 4000 years ago” [REDUNDANT]; **Agreed. Action taken: Removed**
Line 71 – DELETE “to particular areas” (REDUNDANT); “facilitating” would be better than “easing”; **Agreed. Action taken: “to particular areas” removed and “easing” changed to “facilitating”.**
Line 90 – “windthrow” is the technical term for the uprooting and overthrowing of trees by wind; **Agreed. Action taken: changed “windblow” to “windthrow”.**
Line 98 – “… chalkland areas THAT contain …”; **Agreed. Action taken: added “that”.**
Line 116 – DELETE “to” before “towards”, i.e. “… skewed towards Neolithic …”; **Agreed. Action taken: “to” deleted.**
Line 119 – “… Blick Mead WAS a persistent place …” [past tense]; **Agreed. Action taken: added “was”.**
Line 122 – Missing “a” before “unique”; **Agreed. Action taken: added “a”.**
Line 125 – “… high INCIDENCE of auroch …” (not “prominence”); **Agreed. Action taken: “prominence” changed to “incidence”.**
Line 128 – DELETE “particular” (REDUNDANT); **Agreed. Action taken: “particular” deleted.**
Line 146 – INSERT definite article (the) before “later medieval”; **Agreed. Action taken: “the” added.**
Line 155 – “The zoological assessment … identified 271 bone fragments”. The meaning is unclear. This would be better written as, “Of 2430 bone fragments recovered, only 271 were identifiable to species (S3 Table) …” **Agreed. Action taken: rewritten as “Of 2430 bone fragments recovered, only 271 were identifiable to species in the zoological assessment [2] (S3 Table)”.**
Line 159-160 – “… auroch WAS the only animal …”; **Agreed. Action taken: added “was”.**
Line 162 – “Red deer WAS the second …”; **Agreed. Action taken: added “was”.**
Line 163 – Italicise “Sus scrofa”; **Agreed. Action taken: italicised.**
Line 171 – “salmonid and pike” are common nouns, therefore not capitalised; **Agreed. Action taken: capitalisation of both words removed.**
Line 172 – Change “fauna” to “microfauna”; **Agreed. Action taken: changed to “microfauna”.**
Line 184 – “Lathrobium sp.” – “sp.” should not be italicised; **Agreed. Action taken: “sp” no longer italicised.**
Line 203-204 – modify text to read, “… full suite of C14 and OSL dates can be seen …”; **Agreed. Action taken: Text changed as suggested.**
Line 189-190 – “Taken together the insects INDICATE …” (present tense) **Agreed. Action taken: “indicated” changed to “indicate”.**
Line 207 – “Late Pleistocene”. Capitalise “Late”; **Agreed. Action taken: Capitalised “late”.**
Line 211-212 – “… demonstrating evidence of no obvious, major contamination of DNA results …”. Shorten this to, “… demonstrating no obvious contamination of DNA results …” **Agreed. Action taken: Text changed as suggested.**
Line 233-234 – misspelling of Salicaceae; Delete “only”, thus: “…due to very low template sedaDNA…”. **Agreed. Action taken: Spelling error “Saliceae” corrected to “Salicaceae” and “only” deleted.**
Line 285 – “… the data suggest …”. “data” is plural. **Agreed. Action taken: “suggests” changed to “suggest”.**
Line 289 – “nutrient-enriched” [hyphenated] **Agreed. Action taken: hyphenated “nutrient enriched”.**
Line 292 – “Between the depths of 67.82-67.98 m …”. This would read better as, “Between 67.82-67.98 depth, …”. **Agreed. Action taken: Text changed as suggested.**
Line 294 – “high-stress” and “so-called” [hyphenated] **Agreed. Action taken: hyphenated “high stress” and “so called”.**
Line 302 – DELETE “possible”. **Agreed. Action taken: “possible” deleted.**
Line 314 – “…inhibited THE expansion …”; **Agreed. Action taken: “the” added.**
Line 346 – DELETE “of time”. **Agreed. Action taken: “of time” deleted.**
Line 351 – “closed-canopy” [hyphenated] **Agreed. Action taken: “closed canopy” hyphenated.**
Line 358 – “mineral-rich” [hyphenated]; “favourable” [British spelling, for consistency]; **Agreed. Action taken: “mineral rich” hyphenated and “favorable” changed to “favourable”.**
Line 372 – REPLACE “is” with “it”; **Agreed. Action taken: “is” changed to “it”.**
Line 374 – CHANGE “…had to interact with …” TO “… interacted with …”; **Agreed. Action taken: Text changed as suggested.**
Line 388 – “wet woodland” [not hyphenated]; **Agreed. Action taken: hyphen removed from “wet-woodland”.**
Line 390-391 – INSERT “from” between “that” and “Blick”, and DELETE “that” at beginning of line 391. **Agreed. Action taken: Text changed as suggested.**
Line 392 – DELETE “fairly” [REDUNDANT]; **Agreed. Action taken: “fairly” deleted.**
Line 397 – DELETE “as a whole” [REDUNDANT]; **Agreed. Action taken: ”as a whole” deleted.**
Line 406 – “favourable” [BRITISH spelling, for consistency]; **Agreed. Action taken: “favourable” changed to “favourable”.**
Line 410 – “land-use” [adjectival, therefore hyphenated]; **Agreed. Action taken: “land use” hyphenated.**
Line 411 – “19th-century” [hyphenated]; **Agreed. Action taken: “19^th^ century” hyphenated.**
Line 418 – REPLACE “It is likely that” WITH “Likely,”; **Agreed. Action taken: Text changed as suggested.**
Line 424 – redundant “that” [that that]; **Agreed. Action taken: “that” removed.**
Line 434 – “THE sampling procedure …”; **Agreed. Action taken: “the” added.**
Line 438 – “…SEALED with duct tape …”; **Agreed. Action taken: “taped” changed to “sealed”.**
Line 440 – “… THE risk of contamination …”; **Agreed. Action taken: “the” added.**
Line 455 – “THE resulting barcodes …”; **Agreed. Action taken: “the” added.**
Line 471 – DELETE “also”; **Agreed. Action taken: “also” deleted.**
Line 484 – Further methodological details are included in THE S2 Text. [n.b. missing definite article]; **Agreed. Action taken: “the” added.**
